# Electronic Tongues and Noses: A General Overview

**DOI:** 10.3390/bios14040190

**Published:** 2024-04-13

**Authors:** Diego Tibaduiza, Maribel Anaya, Johan Gómez, Juan Sarmiento, Maria Perez, Cristhian Lara, Johan Ruiz, Nicolas Osorio, Katerin Rodriguez, Isaac Hernandez, Carlos Sanchez

**Affiliations:** 1 Departamento de Ingeniería Eléctrica y Electrónica, Universidad Nacional de Colombia, Bogotá 111321, Colombia; manaya@unal.edu.co (M.A.); jgomezbo@unal.edu.co (J.G.); jusarmientoa@unal.edu.co (J.S.); mperezvar@unal.edu.co (M.P.); cflarae@unal.edu.co (C.L.); jruizar@unal.edu.co (J.R.); niosoriog@unal.edu.co (N.O.); ishernandezv@unal.edu.co (I.H.); cesanchezd@unal.edu.co (C.S.); 2 Departamento de Ingeniería Química y Ambiental, Universidad Nacional de Colombia, Bogotá 111321, Colombia; krodriguezar@unal.edu.co

**Keywords:** electronic nose, electronic tongue, applications, trends

## Abstract

As technology advances, electronic tongues and noses are becoming increasingly important in various industries. These devices can accurately detect and identify different substances and gases based on their chemical composition. This can be incredibly useful in fields such as environmental monitoring and industrial food applications, where the quality and safety of products or ecosystems should be ensured through a precise analysis. Traditionally, this task is performed by an expert panel or by using laboratory tests but sometimes becomes a bottleneck because of time and other human factors that can be solved with technologies such as the provided by electronic tongue and nose devices. Additionally, these devices can be used in medical diagnosis, quality monitoring, and even in the automotive industry to detect gas leaks. The possibilities are endless, and as these technologies continue to improve, they will undoubtedly play an increasingly important role in improving our lives and ensuring our safety. Because of the multiple applications and developments in this field in the last years, this work will present an overview of the electronic tongues and noses from the point of view of the approaches developed and the methodologies used in the data analysis and steps to this aim. In the same manner, this work shows some of the applications that can be found in the use of these devices and ends with some conclusions about the current state of these technologies.

## 1. Introduction

Sensors play a crucial role in the industry as they help to monitor and measure various parameters such as temperature, pressure [1], vibration, and humidity, among others [2,3]. Sensors provide real-time data that can be used to know the current state of a process, forecast variables [4], optimize processes, and improve efficiency in manufacturing plants, monitoring systems, and other processes where operational and environmental conditions can change [5,6]. By collecting and analyzing these data from sensors, businesses can identify potential problems before they occur, preventing costly downtime and reducing maintenance costs. Additionally, sensors can be used to monitor environmental conditions and ensure compliance with regulations. This is a really important feature because it opens the possibility to control and monitor variables in real time, avoiding long periods caused by in situ human inspections [7].

Overall, sensors are an essential component of industrial and modern processes, enabling businesses to operate more efficiently and effectively. Multiple sensors are currently used according to the need and the process of monitoring. To analyze these data in most cases, it is necessary to use multivariable techniques and sensor data fusion strategies.

Among a large number of sensors commercially available, gas sensors and sensors that can be submerged in liquids such as screen-printed electrodes have gained relevance and are the basis for electronic tongue and electronic nose systems. These have emerged as a promising technology for electrochemical analysis [8], offering a range of applications in industries such as food and beverage production, pharmaceuticals, and environmental monitoring [9]. These devices rely on advanced sensors to detect and analyze the chemical composition of substances and gases, providing accurate and real-time measurements. By using electronic tongues, companies can ensure that their products are consistent and meet certain quality standards. Additionally, these devices can be used to identify potential contaminants or adulterants, which can help prevent costly recalls or other issues. Overall, electronic tongues are a valuable tool for any industry that relies on precise and accurate analysis of chemical compositions.

The sensors used in electronic tongues and noses are typically based on different principles, including electrochemical, optical, and piezoelectricity. Electrochemical sensors work by detecting changes in voltage or current caused by chemical reactions between the analyte and the sensing electrode. Optical sensors use light to detect changes in the absorption, fluorescence, or reflectance properties of the analyte. Piezoelectric sensors, on the other hand, use a crystal that vibrates when exposed to the analyte, generating an electrical signal that can be measured [10].

Some examples of specific sensors used in electronic tongues and noses include pH sensors, ion-selective electrodes, and gas sensors. These sensors are designed to detect specific chemical properties of the analyte, such as acidity, ionic strength, or gas concentration. Overall, the choice of sensors used in electronic tongues and noses depends on the specific application and the properties of the analyte being tested. By using multiple sensors based on different principles, electronic tongues and noses can provide a comprehensive analysis of the taste, odor, or composition of a sample. It is common that developments in the literature can be found separately for electronic tongue applications and electronic nose applications in specific processes; however, some applications make use of both systems to improve analysis, as will be shown in the next sections.

These technologies have gained importance in recent years because of the advantages in hardware and software, which are important for the acquisition and analysis of information. Some advantages of electronic tongues and noses include the following:

- Precision: Electronic tongues and noses can detect subtle differences in liquids and gases that are not easily detectable by humans, which is fundamental for monitoring and classification processes.
- Time-saving and cost-effective: Because of the resources used in hardware and software, it is possible to acquire a large quantity of data and process it in a short period of time. In some cases, online monitoring is applicable, and communication capabilities are also a possibility for remote monitoring.
- Reduction of human interventions: These systems can be used in an automated way, which results in the benefits of avoiding human bias and opens the possibilities for monitoring processes or environments where humans are not capable of working, such as scenarios where contaminants or environmental conditions are not safe for humans.
- These are non-destructive methods: They do not require the destruction of the samples for analysis because they can interact with liquids and gases without a big particular configuration.
- Scalability: These systems are easily re-configurable in hardware and software, which allows their use in different applications.

Some disadvantages of electronic tongues and noses include the following:

- Costs: These devices can be expensive in some cases because of the initial investment and the maintenance process. However, some multiple commercial solutions and own solutions offer low-cost developments with excellent results. In industrial applications, these costs are small compared with the results that can be obtained.
- Standardization: Currently, there is no standardized method, which results in different solutions depending on how data are analyzed.
- Human testers are required: In spite of the valuable contributions to automating the process where these devices are used, it is necessary to contrast the results with human testers, especially in the alimentary industry.
- Both devices are susceptible to interferences, which has an impact on the final result. As an example, in the case of gas sensors, these interferences can affect the detection of some gases [11].

This work presents a general overview of electronic tongues and electronic noses, starting with some general concepts about these devices and showing the steps in data acquisition and analysis. Some important works may not be covered in this overview due to the vast quantity of literature available. However, this paper aims to present a general overview by addressing concepts about sensors, data acquisition systems, pre-processing, feature extraction, and techniques used for data analysis, machine learning, and AI-based algorithms in both devices. This overview also includes information about some areas where they are used. Conclusions about the information in the review are also included to summarize the relevance of these devices in different areas.

## 2. Electronic Tongues and Electronic Noses

Throughout history, human senses have been used as a quality factor in the alimentary industry and daily activities. Two examples are the gustatory and olfactory senses, which provide information using smell and taste. The sense of taste allows the detection of basic tastes of sweet, sour, salty, bitter, and umami, while the olfactory sense is responsible for detecting the aromas and flavors of food and other elements. These two senses work together in a natural way to create a complete sensory experience when consuming food and beverages. From the point of view of electronics, using sensors to mimic these abilities adds value to systems in the automation of processes such as quality control in several industries such as pharmaceutical [12] and alimentary, among others [13]. It implies the use of sensors that can interact with liquids and gases to provide useful information that can be analyzed with a determined aim. The idea of the use of some of these systems was introduced in the literature in the early 1980s [14,15].

Electronic tongues and electronic noses are sensory devices that mimic the human senses of taste and smell, respectively. They use advanced technology, such as sensors, advanced signal processing steps, and machine learning algorithms, to detect and analyze complex chemical and biological compounds in various substances or gases [16]. The key of these devices is the use of a sensor array [15] because it allows the acquisition of multiple information from the samples for analysis. Electronic tongues and noses have a wide range of applications, including food and beverage quality control, environmental monitoring, and medical diagnosis [17]. They are also used in drug delivery and therapy [18], the development of perfumes, and other products that require detailed analysis of taste and odor.

**Figure 1:**
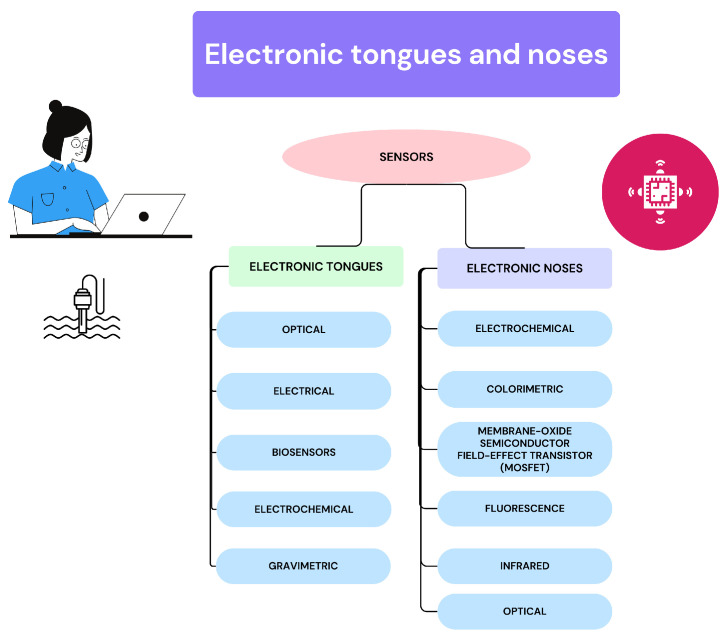
Types of sensors used in electronic tongues and noses.

Figure 1 shows the type of sensors available electronic tongues and noses. As it is shown in the figure, there are some common type of sensors, but each type of sensor makes use of different ways to interact with the liquid substance or the gas. Some of the most used are the electrochemical sensors, where electrochemical techniques are required for this interaction [19].

### How do These Systems Work?

**Figure 2:**
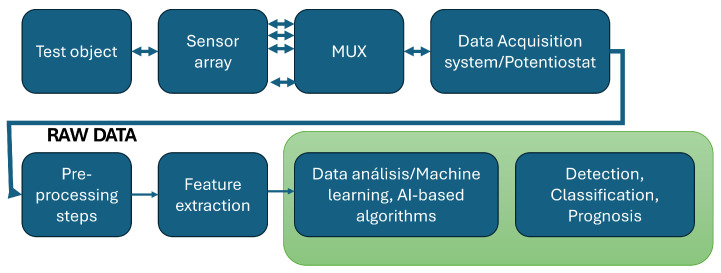
Data acquisition and analysis steps.

Although both sensors allow for the capture of data, the type of information and the way they work are different. While electronic tongues capture information from liquids, electronic noses capture information from gases. Both work as an array of sensors that can detect different chemical compounds or properties in liquids for the case of electronic tongues and gases for the case of electronic noses. Figure 2 shows a general flow diagram of the steps in the work with these devices. During the data acquisition, sensors interact with the liquid substance or gas by using the sensor array. A multiplexor is required to handle the number of sensors, and the data acquisition system allows for the acquisition and collection of information from each sensor.

During the data analysis setup, the multivariate raw data captured by the DAQ system is pre-processed by using different strategies [20], some of which include unfolding, normalization, and filtering. Feature extraction is performed to provide information for the data analysis, which results in the detection of components, classification, and prognosis strategies by using machine learning and AI-based algorithms.

The next sections will address some developments and the different elements according to the steps shown in Figure 2.

## 3. Sensor Arrays and Electrochemical Techniques Used for Electronic Tongues and Noses

Electrochemical techniques are a fascinating area of study from the point of view of automation and electronics. They involve the use of electrical currents to manipulate and analyze chemical reactions and currently are a powerful analytical technique [21]. These techniques have many applications [22], from pharmaceutical quality to environmental monitoring. One particularly interesting application is in the field of biosensors, where electrochemical techniques can be used to detect and measure biological molecules. Overall, electrochemical techniques are a powerful tool for researchers and engineers who are interested in understanding and controlling chemical reactions at a fundamental level.

**Figure 3:**
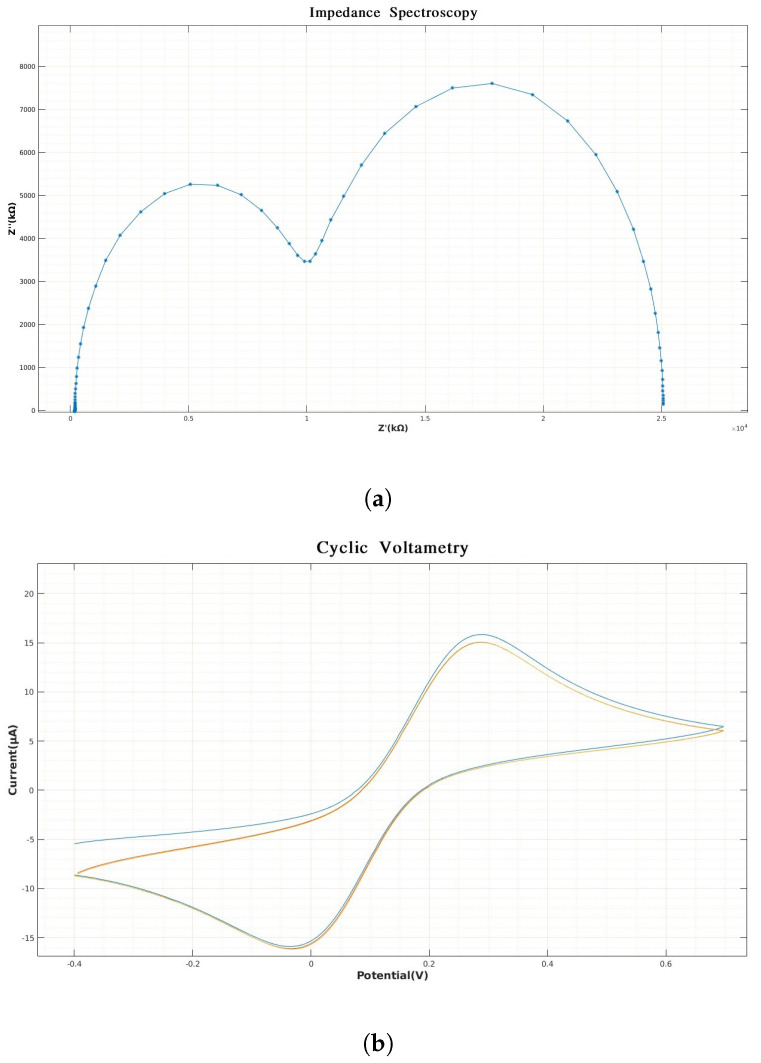
(**a**,**b**) Two ways to represent the electrochemical phenomena, where (**a**) shows the information as a function of a complex number and (**b**) expresses it as a correlation as a function of a given potential expressed in volts. (**a**) Representation of an electrochemical phenomenon in the Nyquist plane using electrochemical impedance spectroscopy (EIS) technique. (**b**) Variation of the electric current as a function of the given potential in a typical oxidation–reduction phenomenon; this technique is known as cyclic voltammetry.

Figure 3 present some examples of the data obtained by using some of the electrochemical methods in electronic tongues. These techniques are commonly applied through a potentiostat that can be used as a system to interact with the sensors. A potentiostat is designed to apply voltammetric, pulsed, amperometric, and galvanostatic techniques, among others [23]. There are multiple devices on the market that allow the application of electrochemical analysis [24,25]. However, most of the commercial devices provide some basic tools to plot and visualize the information from the sensors. Additional analysis and the use of strategies to obtain more information from the data is still an open area for research.

In the case of electrochemical impedance spectroscopy, the phenomenon being measured can present capacitive or inductive behavior, or a combination of both. Figure 3a shows the behavior of a capacitive phenomenon. This is because the graph is located in the positive quadrant of the imaginary axis. In the Nyquist plane, it is also possible to identify the frequency range that describes the behavior of the analyte.

In a capacitive curve, the maximum value of capacitance is given by:

(1) $$C=\frac{1}{Rw_{\left(max\right)}}$$

where $w_{max}$ is the maximum frequency of the system and is chosen by design. $R=Z_{R}$ represents the real value of the impedances when $Z_{j}$ is maximum.

**Figure 4:**
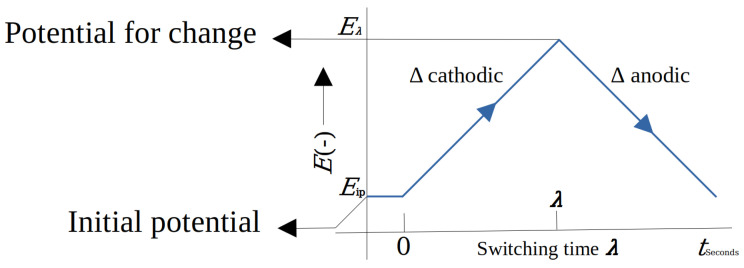
Representation of the potential applied to an electrochemical cell using cyclic voltammetry.

Cyclic voltammetry (Figure 3b) represents the mechanisms that describe some processes of oxidation–reduction or the redox reaction (in this reaction, the oxidizing substance loses electrons and the reducing substance gains electrons), such as organic or metallic systems with organic compounds. Cyclic voltammetry expresses the speed with which this process occurs. By applying a potential between a cathodic and anodic range, intermediate compounds can be obtained as the oxidation–reduction phenomenon is generated. However, not all chemical reactions present the same redox phenomenon, so if a solution only presents the reduction phenomenon, the curve in Figure 3b will only show the upper continuous part. In the same way, if only an oxidation phenomenon occurs, only the lower continuous part of the curve in Figure 3b would be displayed, and these systems are known to be irreversible. The variation between the cathodic potential (initial potential) towards the change potential and the anode potential in a cyclical manner is one of the properties that characterize cyclic voltammetry. Figure 4 shows an example of the potential applied to an electrochemical cell in cyclic voltammetry. This type of signal is known as the excitation signal and expresses the scan rate at which the system is excited. Its speed is normally given in millivolts per second.

**Figure 5:**
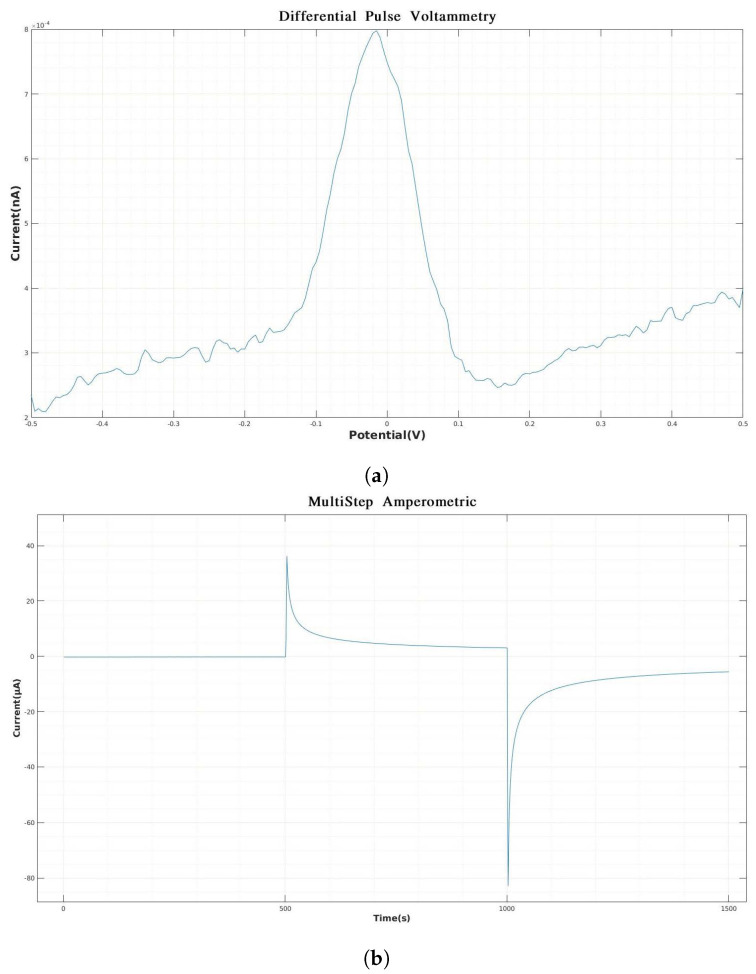
Electrochemical techniques. (**a**) Response obtained by a sensor using a pulsed sweep signal in a period of small as excitation signal. (**b**) Time response of an oxidation–reduction system when a pulsed potential signal is applied.

Chemometric analysis carried out with information obtained from a sensor network is one of the characteristics of electronic tongues. Qualitative analyses of the phenomena that occur at each of the electrodes of the sensor network in an electronic tongue are omitted unless they are presented as an emerging phenomenon that has statistical relevance. Other electrochemical techniques that could be used in electronic languages are shown in Figure 5.

**Figure 6:**
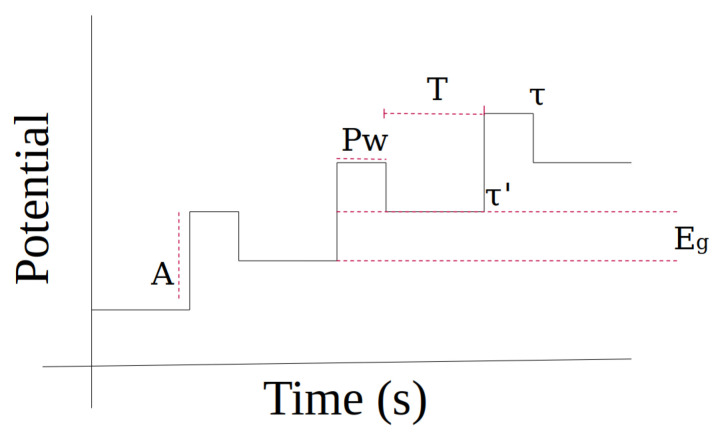
Excitation signal corresponding to differential pulse voltammetry.

Figure 5a is also a type of voltammetry technique, same as Figure 3b. Their difference lies in the signal that excites the system, and this topic will be discussed in depth later. The response in Figure 5b is known as the chronoamperometry curve because its response is given in time. However, the excitation signal is also a pulsed potential signal with a very short time sweep. In the case of differential pulse voltammetry, Figure 5a, a specific potential pulse is imposed for a given time. The trailing edge of said potential does not settle at the initial value but stops at a new value, where a new pulse begins. Figure 6 represents the excitation signal of the differential voltammetry.

This signal has a greater number of parameters that can be configured, such as amplitude (A), pulse width (PW), increase in potential $E_{g}$, period (T), or current sampling time $\left(\tau ,\tau^{\prime }\right)$.

**Figure 7:**
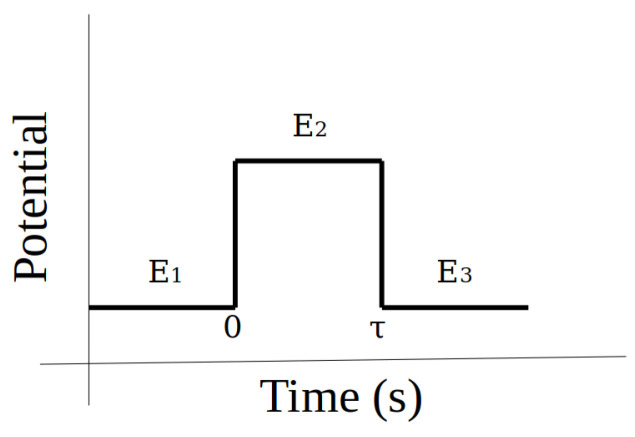
Excitation signal corresponding to the electrochemical chronoamperometry technique.

To obtain the signal response shown in Figure 5b, a pulse like the one shown in Figure 7 is applied. The oxidation and reduction can be also associated to the changes in the electric current.

The responses in Figure 3 and Figure 5 are an example of the response of each sensor. Since the system works as a set of sensors, multiple responses such as the one presented in each figure must be analyzed and preprocessed. Feature extraction and data analysis strategies are required to extract important information from a pattern-recognition point of view.

**Figure 8:**
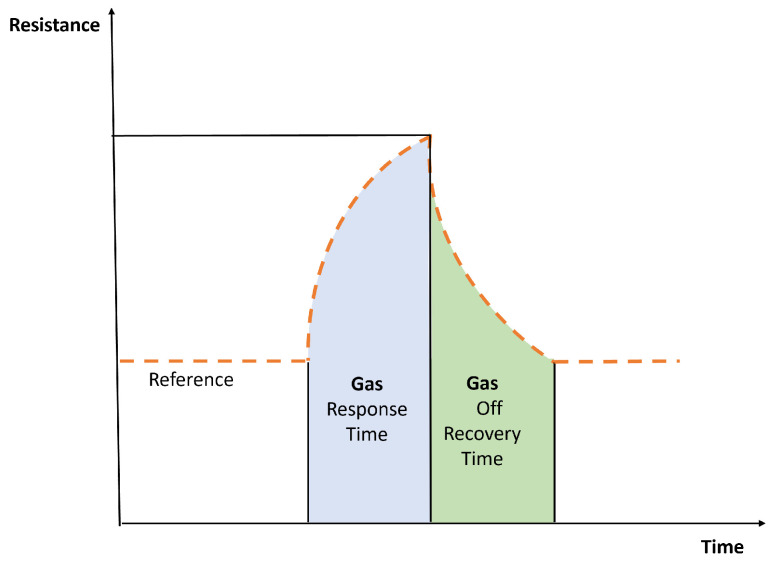
Type of response obtained by sensors in the electronic nose [26].

In the case of the electronic nose sensors, the response by each sensor is a little different. Figure 8 shows a typical response of one MOS or CP sensor used in an array of an electronic nose. This response considers some time response and recovery associated to each sensor. As in the case of electronic tongues, the raw data requires some additional steps to obtain relevant information [26].

### 3.1. Electrochemical Sensors

Electrochemical sensors are the largest group of chemical sensors and are characterized by their operation based on the relationship between electricity and chemical reactions, which refers to the reaction between the electrode and the analyte [27]. They are divided into three main subtypes: potentiometric, amperometric, and conductimetric [28]. Electrochemistry involves the transfer of charges to another phase through electrodes, whose reactions are chemically modulated and are the basis of the sensing process of these elements [28]. Thus, it is possible to speak of chemical sensors when the sensing element of the electrode is organic or inorganic so that it has a selective interaction with the substance to be analyzed. This type of sensor, therefore, detects a certain biological or chemical reaction with the material to be studied, called the analyte, through the receptors and transforms it into a primary signal of another type (electrical, optical, or thermal, among others) using some type of transducer, whose main characteristics correspond to the specificity in its interaction with the compound of interest, its high sensitivity, short analysis time, and versatility [29]. It is important to remember that the difference between sensors and transducers corresponds to the fact that the former are always in contact with the instrumentation variable, so they take advantage of the properties of the material in which they are located to quantify some characteristic of interest; while the latter use different methods to convert the data received into useful information for study by means of an electronic or computer system [30], providing information about the sample, the process, or the system to be investigated.

There are three fundamental rules for all electrochemical sensors, regardless of their type or configuration, as follows:

1. It requires the circuit to be closed. In other words, it needs at least two electrodes in a single electrochemical cell [28]. Electrically, these are usually called the working electrode (sensor), where the electrochemical reaction to be studied takes place, and the reference electrode (return signal), which controls the variable of interest on the sensor.
2. The electroneutrality condition, which states that the sum of negative charges in an electrochemical cell must be equal to the sum of positive charges, must be met.
3. The charge transport in the sample can be electronic or mixed (electronic–ionic) [28], which corresponds to an essential aspect to the sensor performance. If a non-zero current (charge passing) flows through the mixed interface, electrolysis occurs according to Faraday’s law, where electronic conduction in the metal is converted to ionic conduction in the sample.

Table 1 summarizes the operation of the three main types of electrochemical sensors, considering that there are a large number of classification categories [31].

The two most important factors in the performance of electrochemical sensors are the resistance to charge transfer and the exchange current density [28]. Since the current–voltage curve is affected by the nature and concentration of the electroactive species, it is essential to ensure that the electrode surface has an adequate surface area and high electrical conductivity [37], allowing rapid transfer that makes detection fast and accurate.

### 3.2. Some Materials Used in Electrochemical Sensors

The materials used in the design of electrochemical sensors are strongly associated with a specific area of study; however, by taking a redox process as the base electrochemical phenomenon, some materials present a greater or lesser contribution of electrons to said process. Some of these materials are palladium, platinum, and alloys such as gold–silver, nickel, gold, or tungsten, among others. The material selected for a type of electrode depends on the speed with which the electrochemical phenomenon is to be measured.

#### 3.2.1. Carbon-Based Sensors

The construction of electrodes with high sensitivity values involves the additive manufacturing of sensors where the high costs in the implementation of materials with gold or nickel alloys raise the production costs, so the carbon-based additive manufacturing [38], such as carbon or graphene mixed with thermoplastic materials such as polylactic acid (PLA), significantly reduces the complexity of the process and its production costs. One of the applications where the sensor with carbon-based electrodes presents significant advantages is in the detection of carbon dioxide [39] $CO_{2}$ in internal combustion vehicles, where high temperatures present a significant challenge. In these sensors, a cell with a carbonate electrolyte is commonly used in the detection and reference electrodes, with a mixture of carbonates.

Another type of carbon-based electrodes are those called vitreous carbon [40] or glass-like carbon. This type of electrode is formed by the carbonization of phenol-formaldehyde resin, arranged in a hexagonal pattern. This type of material provides a relatively high current density and can be produced in the form of films, sheets, or powder. There are also screen-printed carbon-based electrodes [41] (SPCE). Their big difference with glassy carbon electrodes is their disposable nature and low manufacturing cost.

#### 3.2.2. Graphene-Based Electrodes

Graphene is a material that has provided significant advantages to electrochemical sensors, due to its relative high electrical conductivity and low physical density. Some of its applications are in the detection of dopamine [42], where this material is used in form of graphite paste, graphene sheets, or graphene ink. Sensors based on graphene electrodes can also be designed as biosensors using the chemical vapor dispersion method [43] on nickel or copper substrates or by developing graphene oxide [44], through which graphene structures with a lateral dimension of less than 1 µm and a thickness of 2.8 ± 0.2 nm are fabricated.

### 3.3. Two-Dimensional Materials-Based Electronic Noses

Some of the materials used in electronic noses are conductive polymers, metal oxide semiconductors, and quartz crystal microbalance.

Graphene is probably the most widely used 2D material for making gas sensors. An overview can be made for incorporating 2D materials in functional devices. One of the methods of implementing graphene (bottom-up) involves organic synthesis and chemical vapor deposition. This process leads to thin plates of graphene and other 2D materials, which have shown great compatibility with SI-CMOS technology [45].

Speaking about materials, electronic noses can also help in sample enrichment methods, one of them being “purge and trap”. This method involves passing an inert gas through a substance that can exist as a gas, liquid, or solid. The volatile components in the substance are then captured by a specific adsorbent trap. The trap is then heated, causing the captured molecules to be released and made available for measurement [46]. The most important feature of this method is the load capacity of the trap, because if the trap is saturated, it can break the volatiles. This overloading can be avoided if the trapping material is carefully chosen [47]. Electronic noses can be used to determine these materials, as it will help if the experiments and materials created with this technique are made to have the highest possible success rate. Moreover, they can increase the efficiency of cross-selective sensor arrays for gas analysis [48]. In gas sensing applications, MXenes, which belong to the 2D structures, were discovered in 2011. They are molecular sheets obtained from carbides and nitrides of transition metals. MXenes are currently widely implemented due to their high surface-to-volume ratio and high electrical conductivity, enabling a high gas adsorption and tuning of surface chemistry and functional groups [49]. Additionally, chalcogenides are applied in sensing devices. They consist of a chalcogen anion (elements from group 16 of the periodic table, generally sulfides, selenides, and tellurides) and a more electropositive element [50].

#### 3.3.1. Molecularly Imprinted Polymer (MIP)-Based Electrodes

MIP electrodes consist of printing a synthetic material on a substrate with cavities that specifically adapt to the anite for which they were designed [51].

MIP-based electrodes have high selectivity, which is why they are widely used in separation processes. One of the most important developments has been achieved by applying an MIP to a nanostructured carbon material surface [52]. This is due to its catalytic properties in redox reactions and improvements in voltammetric response, additionally providing important improvements in identifying an analyte in complex solutions. MIPs can also be found in graphene nanostructures to improve the detection sensitivity of the target analyte [51]. They can also be integrated with metal electrodes [53] in neuroscience applications using techniques such as differential pulse voltammetry. An example of this is printed polymeric nanobeads [54] for the detection of sarcosine (SAR) in urine. In general, MIP sensors are excellent for the detection of biomarkers. MIP electrodes also have applications in the detection of contaminants in foods where they are known as MIP electrochemical chemosensors.

With the advancement of technology, the development of new techniques has been achieved that allow different types of materials to be efficiently integrated, as is the case of electrodes made with particles of materials in two-dimensional layers (2DLM) [55], where they use materials such as graphene and its derivatives, MXenes, phosphorene, transition metal dichalcogenides, and organometallic structures. In this same direction are nanostructured materials such as those based on CMOS technology [56], the detection of analytes through fluorescence, as well as electrode sensors based on iron, cerium, or mercury [57].

The following section will address in more depth the components that are at the forefront of the development of highly sensitive electrodes for electrochemical sensors.

#### 3.3.2. New Emerging Materials

The current development of electrochemical sensors emphasizes miniaturization, heightened sensitivity and enhanced biocompatibility of materials, efficient detection of specific analytes, and selective determination of natural and biological components, contaminants, and additives. [58]. In general, the electrochemical sensors can be modified and tailored involving nanocomposites, conducting polymers, biosensors, and nanoparticles. Nevertheless, the discovery and combination of new materials challenge a conventional classification, as materials are progressively being intermixed. Among the trends in the development of sensors is the use of biosensors, taking advantage of the biological interactions and responses that can be converted into an electrical signal, providing high sensitivity, lower cost, and versatility in various applications such as healthcare, environmental monitoring, and food [59,60]. Table 2 summarizes some of the materials used.

## 4. Pre-Processing and Feature Extraction

Preprocessing is a crucial step in the context of an electronic tongue before applying classification or machine learning algorithms because it helps enhance the quality of the input data by reducing noise, normalizing variables, and removing irrelevant information. Electronic tongues and noses often generate complex, multi-dimensional data from the array of sensors. Preprocessing techniques like unfolding, noise reduction, normalization, and scaling ensure that the data is in a suitable form for analysis, improving the performance and interpretability of the subsequent classification or machine learning algorithms [75]. This step aids in extracting meaningful patterns, enhancing the accuracy of taste analysis, and enabling more effective discrimination and identification of different substances from the point of view of multivariate analysis.

### 4.1. Dimensionality Reduction

The use of a dimensionality reduction technique is essential for data analysis when using electronic tongues and noses because of the complexity of datasets obtained by the sensor array. The combination of both systems can be an ideal step for applying sensor data fusion strategies. This step aims to transform high-dimensional data into lower-dimensional data without losing essential information by identifying relevant features that capture the essential characteristics of data [76]. Some of the strategies used in these systems include Principal Component Analysis (PCA) [77,78], Kernel principal component analysis [7], project pursuit [79], isomap [7], Laplacian Eigenmaps, Locally Linear Embedding (LLE), modified LLE [79], Hesian LLE [80], t-distributed Stochastic Neighbor Embedding (t-SNE) [79], and Linear Discriminant Analysis (LDA) [77,81], among others.

### 4.2. From the Time Domain to the Frequency Domain

The use of some techniques such as the Fast Fourier Transform or the wavelet transform [82,83] has also been addressed in the work with these devices. These techniques are useful to extract features by changing the original time-domain data to frequency-domain data [84].

## 5. Data Analysis

Following data preprocessing and dimensionality reduction, the subsequent step in data analysis involves an automated learning process. In terms of learning strategies, these processes can be categorized into three main groups: supervised, semi-supervised, and unsupervised learning. Supervised learning algorithms are a specific category of machine learning algorithms where the model is trained on a labeled dataset. These algorithms learn from the labeled data to make predictions or decisions on unseen data. Some of the most known algorithms are decision trees, neural networks, Naïve Bayes, partial least squares (PLS), k-nearest neighbors (KNN), and support vector machines (SVM). Supervised learning algorithms are commonly used for classification and regression tasks in electronic tongues and noses. Their objective is to predict the class or value of a target variable based on input features. In contrast to supervised learning algorithms, unsupervised learning algorithms are trained on unlabeled data, meaning that there are no corresponding target labels for the input data points. These algorithms learn patterns and structures from the input data without explicit supervision. Some of the most used are Principal Component Analysis (PCA), Kernel PCA, t-Distributed Stochastic Neighbor Embedding (t-SNE), Laplacian Eigenmaps, and Locally Linear Embedding (LLE). They are commonly used for clustering, dimensionality reduction, and anomaly detection tasks in electronic tongues and noses, where the objective is to uncover hidden patterns or structures in the data.

To perform the aforementioned analyses, various multivariate techniques have been developed and can be broadly classified into two main categories. The first category comprises chemometric statistical techniques, while the second encompasses various artificial neural networks (ANN).

Numerous comparative studies have been conducted to determine the most effective technique for pattern recognition and multivariate analysis using electronic tongues and noses. The learning algorithms that are usually used include Decision Trees, Neural Networks, Naïve Bayes, PLS [85], KNN, SVM, SFA, PLS-DA, DFA, SLDA, and Rule learners [76]. Table 3 shows some of the applications and algorithms used for data analysis during the last year as an example to show the big quantity of approaches and applications in electronic tongues and noses.

## 6. Applications of Electronic Tongues and Electronic Noses

Electronic tongues, or e-tongues, are becoming indispensable to the industry and to monitoring systems. Their applications cover several fields, some of which still need to be explored. Generally, they are used for quality analysis and verification of food, beverages, dairy products, pharmaceutical products, and cosmetics. Moreover, there are investigations around using e-tongues in disease detection and checking the state of the food [96].

On the other hand, electronic noses or e-noses are sensors with great potential in the perfume and the food industry as well. The combined use of electronic noses and tongues can allow one to obtain a broad information spectrum without biased data. Biases in data can arise due to the use of sensor panels comprised of groups of both trained and untrained individuals. Moreover, people cannot be used for the detection of toxic gases or non-consumable substances [97]. This is a wide area where these sensors, in combination with a proper discrimination algorithm and a machine learning approach, can provide a low-cost, on-site, highly effective, and almost immediate alternative to other traditional qualitative and quantitative analysis techniques. The following subsections show some of the areas where these elements have been used.

### 6.1. Health Care

Various researchers have used the sense of smell to determine the physical condition and the general health of patients throughout history. Many of the diseases produced in humans give off odors that are recognizable in a person’s day-to-day life. Some of these are well identified by the literature and Table 4 shows some of them as explained in [98].

Taking advantage of the human body to produce some specific odors associated to some illnesses, which are relevant, for instance, in diseased body parts or fluids obtained from these tissues, it is possible to identify biomarkers for the detection of volatile organic compounds (VOC). Table 5 shows some of these VOCs according to [98].

One example of how these aromas are used is the use of non-selective gas sensors for the analysis of breath in lung cancer detection [99]. Health care and disease prevention have also used electronic tongues to detect illnesses such as cancer in urine samples. This application is studied due to its practicality, since it is a non-invasive method, and due to its cost-effectiveness compared to other methods used for the same purpose, such as Raman spectroscopy, infrared spectroscopy, and fluorescent spectroscopy. Furthermore, electronic tongues can discriminate, classify, and analyze information with high complexity in a shorter amount of time [96]. Electronic noses have been also used in the analysis of volatile organic compounds such as those that can be found in urine, blood, and exhaled breath [100]. Colorectal cancer detection is other interesting area where electronic noses can detect volatile organic compounds (VOC) [101]. In these applications, the VOCs are used as a non-invasive biomarker for early cancer detection [102]. The combination of electronic tongues and noses has also been explored in the health care area. As an example, these devices were combined for the early detection of liver cirrhosis as a non-invasive method, considering that the effectiveness of both devices increases the early detection [103].

Recently, and as a strategy for COVID-19 detection [104], an array of paper-based electronic tongues has been used, which is friendlier for the user, and employed compounds like nanoparticles, metal ion compounds, and organic dyes as sensing materials that react with human serum samples [105] to detect the presence of acetylsalicylic acid (ASA) in human physiological fluids using a chitosan-based electrochemical sensor and a voltammetric electronic tongue. This approach aims to prevent ASA poisoning. The PCA and DFA methods were used to ease the visualization of the dataset, and the PLS regression method was employed to implement predictive models [106]. This approach was also used for the diagnosis of cancer through saliva using supervised and unsupervised machine learning with a voltammetric e-tongue. This method could determine if a patient had cancer or not, with an accuracy above 80% in the binary discrimination [107]. COVID-19 detection has also been assisted by electric noses. As an example, in [108], the combination of clinical signs and symptoms, laboratory tests, imaging measurements, and an electronic nose was developed as a non-invasive method to rapidly detect COVID-19 by using metal oxide semiconductor gas sensors. The detection considers the VOCs in the exhaled breath and can be used in combination for the classification of patients according to the pulmonary diffusion capacity [109].

Other applications include the palatability evaluation of Traditional Chinese Medicine (TCM) oral formulations including syrup, mixture, oral liquids, tincture, and granules, evaluated by combining human sensory evaluation, e-tongue evaluation, and saliva amount evaluation [110].

### 6.2. Food Industry

e-Tongues and e-noses have also been widely used in research to check the quality, taste, and state of food. For instance, e-tongues have been employed in the qualifying process of the taste of umami in Hanwoo meat [111]; in the comparison between Koji-mold ripened cheese and Camembert cheese flavors, utilizing different strains of Koji for this purpose [112]; for the discrimination of honey based on its botanical origin, using a potentiometric electronic tongue to specifically distinguish between monofloral, polyfloral, and honeydew honey [113]; and in the discrimination between Lager Beer types, considering their electrochemical active compounds, through an array of screen-printed electrodes [114]. Precision agriculture is vital nowadays to produce more food and optimize the growing space. Soil monitoring is fundamental for this purpose. In this way, there have been some developments, such as the proposed microfluidic system based on an array of four Layer-by-Layer sensors made of films deposited onto gold interdigitated electrodes (IDEs) inside a microchannel made of polydimethylsiloxane (PDMS), to discriminate different soil samples rich in sulfur, nitrogen, phosphorus, magnesium, calcium, and potassium. The PCA, Interactive Document Map (IDMAP), and Sammon’s Mapping methods were employed to analyze and discriminate the sensor data [115].

### 6.3. Product Authenticity and Identification

In the market, it is common to find many food products that claim to be authentic or 100% natural but they are imitations or adultered versions of the products with lower quality, which have caused great damage to the trust and health of consumers. In this field, electronic noses and tongues are beginning to be used as tools to strengthen quality controls and be able to guarantee the authenticity of products in a faster but equally reliable way [116]. Most of these works are based on potentiometric and voltammetric multi-sensor devices’ point of view. Some of the processes considered by electronic tongues and noses include the following:

- Authenticity and species identification of Fritillariae cirrhosae [95].
- Determination of synthetic antioxidants in edible olive oils [88].
- Honey authenticity determination [80,117,118,119].
- Detection of Monilia Contamination in Plum and Plum Juice [81].
- Assessment of authenticity and adulteration of sausages [120].
- Detection of characteristics for origin region classification. As an example, tea classification, where fermentation processes and growing conditions are different [121].
- Fruit freshness classification. As an example, Huang et al. [122] developed an electronic nose to classify fruits in three classes—fresh, sub-fresh, and spoiled—using a zynq 7000 SOC and Labview for processing including PCA + LDA and PCA + KFDA to obtain 96.2% accuracy when the system was evaluated with jackfruit, strawberry, Hami melon, pear, apple, grape, and banana. The system takes advantage of the sugars in the fruits that decompose into alcohol by using alcohol gas-sensitive sensors.

### 6.4. Product Quality

Regarding the identification of qualitative features in food and beverages, e-tongues and e-noses as an example have been employed to evaluate the taste characteristics and composition of Italian wines, due to the sensors’ capability to identify components and patterns in complex substances [123]. Similarly, an electronic tongue multi-sensor system (ET) was employed for age estimation and compound identification of Madeira wines. The results indicated that the e-tongues system yielded significant results in estimating the age of the wines [124].

### 6.5. Environmental Applications

The monitoring of environmental conditions is a big area where electronic sensors have multiple applications, allowing a better understanding of ecosystems and the impact of human activities on them. Several developments are available for environmental engineering applications.

#### 6.5.1. Detection of Substances in Water

Water quality monitoring is an important issue nowadays, due to several contaminating agents present in water resources. For this reason, it is crucial to inspect the water’s integrity. Due to the capability to identify several characteristics and discriminate complex substances, electronic tongues have been used to detect substances, the measurements of chemical species in solutions [125], and qualitative features in the water. For instance, electronic tongues have been employed for the detection of taste and odor compounds originating from algae in water, due to their practicality and low cost [126]. The analysis of mineral water was also explored to compare different brands [127]. Moreover, it was proposed the employment of voltammetric e-tongues for the identification and classification of microplastics in water, because electronic tongues can provide a rapid, low-cost, and on-point analysis of components in water with a high accuracy [128]. Microplastic monitoring is important because of the risk in human health [129] associated with ingestion because of bad treatment of water for the food industry [130]. In this sense, the identification and quantification of microplastics in the marine environment have also been explored [131]. Other approaches include the development of systems for quantitative analysis of inorganic ions in aqueous media by using sensor arrays composed by graphite and zeolites to evaluate samples in rivers [132].

#### 6.5.2. Environmental Contaminants Detection

The use of ionic liquids for electrochemical sensors was explored for drug residues, pesticides, and heavy metals [133].

Heavy metals are another polluting agent in water bodies due to anthropogenic activities. They threaten marine ecosystems and public health because the heavy metal ions can accumulate in living beings. There are several investigations in this specific area of study. For instance, through cross-sensitivity analysis, an array of 33 potentiometric electronic tongues made of vitreous and crystalline membranes were used in research where data was analyzed with partial least squares and artificial neural network [134]. Solvent polymeric and chalcogenide glass membranes used as potentiometric sensor arrays to detect ultra-low activities of copper, zinc, lead, and cadmium ions found in seawater have also been used for this aim [135]. Electronic tongues have also been tested with ternary nanocomposites based on cellulose nanowhiskers (CNW), electrospun nanofibers, and silver nanoparticles in their layers. They were set in an array of sensors and submerged in contaminated water with lead, cadmium, nickel, and copper ions [136].

Photoelectric electronic tongues-based colorimetric sensors using pyridyl azo and porphyrin indicators to detect heavy metal ions in fishes commercialized in Zhenjiang, China, is another application that can be found in the detection of heavy metals oriented to the alimentary industry and ecosystem monitoring. In this work, the data analysis and modeling were developed through partial least square regression (PLS) and extreme learning machines (ELM) [137].

Another major contaminant found in water is pesticides, because it is extensively used in agriculture. An electronic nose has been demonstrated to be capable of detecting these substances in groundwater, showing its advantages as a monitoring system. [138]. This kind of contaminant has been also explored with the use of an electronic tongue for soil pesticides pollution detection and specific recognition, showing the advantages of its use in in situ detection [139].

#### 6.5.3. Odor Detection

The authors of [140] showed, for instance, the application of MOX sensors in water quality monitoring, air quality applications, process control, and verification of odor control systems efficiency.

### 6.6. Criminalistics and Rescue

One potential use of electronic noses is in criminal investigations to locate missing persons. The decomposition of corpses is relevant to these types of criminal cases, as it can indicate how long the person has been dead. However, in criminal cases, the body is always hidden. Although detection dogs are currently the most effective tool, they are limited by their cost and operational limits.

In an experiment conducted by Amber Brown and company, together with a corpse donated for research, they programmed a NOS-E type electronic nose to see how it behaved in the decomposition process of the corpse. During the first phase, the corpse was giving off odors around the torso and head that the nose was able to detect without any problem, and in more advanced phases where the head presented a more advanced decomposition than the whole body, the electronic nose had a peak of detection. In summary, the nose was able to detect the body at all stages of decomposition. For more detailed information see [141].

Electronic noses can also be used to detect people who have been kidnapped. The characteristics of the gases that can detect the location of live-trapped persons can be seen in the Table 6.

## 7. Conclusions

Electronic tongues and noses have proven to be invaluable tools in various scenarios due to their ability to accurately and quickly analyze features in liquid substances and gases. An important feature that allows their versatility is the use of sensor networks and specialized data analysis techniques. With the development of better sensors, data acquisition systems, and hardware tools for processing and analyzing data in real time, these systems are expected to tackle more sophisticated applications and developments. Although electronic tongues and noses can interact differently because of the sensors, information analysis follows similar steps. This is an important feature because it allows the use of sensor data fusion strategies. The literature provides examples of applications that combine both systems and confirm that this is an active area for further research. In most cases, dimensionality reduction is necessary because of the multivariate nature of data and to facilitate the use of AI strategies. The most common strategy in the revised works is Principal Component Analysis. However, for identification and classification tasks, multiple strategies are developed, and there is no common method for managing the data.

Electrochemical sensors are essential for the development of this kind of device and continue to be an active research area. This is because of the way they interact and because electronic developments allow the addition of important features for their use. In this sense, it was found that both devices have an essential use as monitoring systems. From our point of view, automated and intelligent electronic tongues and noses will appear shortly as smart devices with online capabilities. Each system has developments in multiple applications, including but not limited to health care, environmental monitoring, criminalistics, and rescue, among others. However, they can be used in all the areas where smell and taste sense are a solution. Some problems related to the use of these devices as a commercial and accepted solution are interferences caused by changes or failures in hardware and changes in operational conditions. This area continues as a problem to solve with the aim of providing robustness to the system.

Although the systems can be used in separate ways, multiple works show that combining these devices improves the analysis of the information captured. In the same way, the trend is to develop a multisensor system similar to the human sense system due to the multiple applications that require a combined analysis to produce a more accurate result.

## Figures and Tables

**Table 1:** Electrochemical sensors.

Electrochemical Sensors		
**Potentiometric**	**Amperometric**	**Conductimetric**
The sensors use two electrodes, a working and a reference electrode [32].	Usually these sensors use a working and reference electrode. An auxiliary electrode is used to complete the electrochemical cell [30,32,33].	Sensors have a configuration of two electrodes of different materials, a working and reference electrode [32], but it can work with two equal electrodes, using an alternating voltage, since the application of a potential difference creates an electric field that orders the movement of the ions. The main advantage of the differential mode is the cancellation of interference [34]
The sensors are based on the potential difference between the working electrode and the reference electrode [32,33]. This potential difference is generated by an ion exchange between the working electrode surface and the analyte [35].	The sensors measure the electrical current generated by the electrocatalytic reaction when a potential is applied between working and reference electrodes [31,33].	These sensors are based on the measurement of conductivity changes at different frequencies [36]
Sensors are generally classified as ion selective electrodes (ISE) and are based on field effect transistors [36].	The measured electrical current is proportional to the analyte concentration [31] and is modeled according to Fick’s law [29,32].	These sensors have been developed using polymers and metal oxides. Biosensors are manufactured by modifying the electrode with biological material [32].

**Table 2:** Materials used for Electrochemical sensors.

Electrochemical Sensors	
Elements	Application
Nanocomposites featuring gold nanoparticles and catechol	Uranyl ion detection, addressing nuclear cycle contamination concerns [61]
Polyaniline-based nanocomposites	Detection of biomolecules and environmental pollutants and can be used to develop supercapacitors [62,63]
Polyaniline, Polypyrrole, Polythiophene, and Poly (3,4-ethylenedioxythiophene)	Are the most frequently used conducting polymers due to their customized and enhanced electrical conductivity by electronic doping, high environmental stability, and biocompatibility [64,65,66,67]. They impart selectivity to the sensors, enabling them to identify particular substances. [68]. Differentiating varieties of coffee and black tea has been achieved using electronic tongues that combine conductive polymers on nickel-immersion-gold electrodes and a copper matrix on layer-by-layer nanostructured materials [69,70].
Carbon, metallic, and metallic oxide nanoparticles	Allow the building of electrodes and assist matrices, with metal oxide nanoparticles offering superior conducting properties [71]. Electrodes based on glassy carbon with different modifying materials detect glucose and tartaric acid in red wine [72]. Combinations of nanoparticles in membranes have shown promise in applications such as wine and milk detection [58]. Detection of toxic substances such as pesticides and disinfectants through metal oxides Fe_3_O_4_, ZnO, Au, CuO, MnO_2_, y TiO_2_.
Graphene oxide plus epoxy resin electrodes, enzymes including different catalysts	Glucose detection [60,73,74]

**Table 3:** Some of the methodologies developed for data analysis of electronic tongues and noses during the last year.

Methodologies		
**Device**	**Application**	**Methodology**
electronic tongue	Detection of bovine mastitis with milk samples	multidimensional projection technique interactive document mapping (IDMAP) + parallel coordinates + Decision Trees [86]
electronic nose	Determination of quality of tea from different picking periods	Adaptive pooling attention mechanism (APAM) (Adaptive multi-scale pooling structure + concatenation + convolutional neural network) [87]
electronic tongue	Detect synthetic antioxidants edible olive oils	Low level data fusion (LLDF), mid-level data fusion (MLDF) + Discrete wavelet transform (DWT) + Artificial neural network (ANN) [88].
electronic nose	prediction of tomato plants infected by fungal pathogens	PCA + Discriminant Functions Analysis (DFA) + Backpropagation neural network (BPNN) [89]
electronic tongue	Liquor beverage classification	unfolding + mean centered group scaling (MCGS) + (Hessian LLE/Isomap, Laplacian Eigenmaps/LLE/LTSA/and modified LLE) + Multi-layered perceptron (MLPN) [90]
electronic nose	Determination of Pitaya quality	Autoscaling + PCA + Linear discriminant analysis [91]
electronic nose	detection and identification of subterranean termites	PCA + Quality factor analysis (QFA) [92]
electronic tongue	classification of honeys	unfolding + mean centered group scaling (MCGS) + (PCA/t-SNE/Laplacian Eigenmaps/Isomap/Locally Linear Embedding(LLE)) + k-nearest Neighbors (KNN) [80]
electronic nose and electronic tongue	prostate cancer detection in exhaled breath and urine samples	Mean-centering function + Orthogonal Signal Correction (OSC) + PCA + Discriminant Function Analysis + (Quadratic discrimination analysis (QDA)/Naïve Bayes/Support Vector Machine (SVM)/k-nearest Neighbors (KNN)/Random Forests/Decision Trees) [93]
electronic tongue + electronic nose	Flavor perception	This work proposes an Olfactory–Taste Synesthesia Model (OTSM) and compares the results with PCA-ELM, WF-LPP, VIP-ELM, VIP-RF, VIP-GA-SVM, BPNN, ELM, GS-SVM, and RBFNN [94]
electronic tongue + electronic nose + electronic eye + near-infrared spectroscopy	Authenticity and species identification of fritillariae cirrhosae	PLS-DA for authenticity and PCA-DA for species authentication [95]

**Table 4:** Descriptive aromas previously used for diagnosing human diseases [98].

Disease	Body Source	Descriptive Aroma
Anaerobic infection	Skin, sweat	Rotten Apples
Bladder infection	Urine	Amine-Like
Gout	Skin	Gouty Odor
Liver failure	Breath	Musty fish, feculent
Pseudomonas infection	Skin, sweat	Grape
Scrofula	Body	Stale Beer
Yellow Fever	Skin	Butcher’s Shop

**Table 5:** Volatile Chemical Biomarkers used for diagnosing human diseases [98].

Disease	Volatile Chemical Biomarkers
Allograft rejection	Carbonyl sulfide
Breast cancer	C4–C20 alkanes
Diabetes	Acetone, ethanol, methyl nitrate
Lung cancer	Alkanes, ketones, specific aromatic hydrocarbons
Necrotizing enterocolitis	2-Ethyl-1-Hexanol
Schizophrenia	Pentane, carbon disulfide

**Table 6:** Application: Location of live-trapped persons [142].

E-Nose Detection	Substrates	Compounds Present	Chemical Classes
Respiration gases	Inorganics	Small mol. wt, gases	Oxides
Stress compounds	Ketosis (Starvation)	Aliphatic HC	Ketone
Wound compounds	Contusions, lacerations	Aliphatic	VOC mixture
Waste excretion	Urination	Aliphatic	Carbamide

## Abbreviations

The following abbreviations are used in this manuscript:

DAQ	Data Acquisition
DWT	Discrete Wavelet Transform
E-Nose	Electronic nose
E-Tongue	Electronic tongue
KNN	K-Nearest Neighbor
LLE	Locally Linear Embedding
MUX	Multiplexor
PCA	Principal Component Analysis
PLS	Partial least squares
PLS-DA	PLs-Discriminant Analysis
QFA	Quality Factor Analysis
TCM	Traditional Chinese Medicine
t-SNE	T-distributed Stochastic Neighbor Embedding
VIP	Variable influence on projection
VOC	Volatile organic compounds
